# Determinants of delayed initiation of breast milk expression among mothers of premature infants in Kelantan, Malaysia

**DOI:** 10.7717/peerj.21159

**Published:** 2026-04-23

**Authors:** Wan Haiera Wan Nor, Noraini Mohamad, Norkhafizah Saddki, Zainab Mat Yudin, Tengku Alina Tengku Ismail, Zaharah Sulaiman

**Affiliations:** 1 Obstetrics and Gynaecology Department, Hospital Raja Perempuan Zainab ll, Kota Bharu, Kelantan, Malaysia; 2 Medical and Basic Dental Sciences Unit, School of Dental Sciences, Health Campus, Universiti Sains Malaysia, Kubang Kerian, Kelantan, Malaysia; 3 Dental Public Health Unit, School of Dental Sciences, Health Campus, Universiti Sains Malaysia, Kubang Kerian, Kelantan, Malaysia; 4 Department of Community Medicine, School of Medical Sciences, Health Campus, Universiti Sains Malaysia, Kubang Kerian, Kelantan, Malaysia; 5 Women’s Health Development Unit, School of Medical Sciences, Health Campus, Universiti Sains Malaysia, Kubang Kerian, Kelantan, Malaysia

**Keywords:** Premature infants, Knowledge, Barriers, Breast milk expression initiation

## Abstract

**Background:**

The initiation of breastfeeding in premature infants is often more challenging than in full-term infants due to their physiological immaturity. These infants often exhibit reduced alertness, limited stamina, and underdeveloped coordination of sucking, swallowing, and breathing, making effective latching difficult. As a result, lactation initiation and maintenance often begin with breast milk expression. This study aimed to determine the prevalence of delayed breast milk expression initiation (beyond six hours after delivery) and to identify factors associated with this delay among mothers of premature infants admitted to the Neonatal Intensive Care Unit (NICU) at two tertiary hospitals in Kelantan, Malaysia.

**Methods:**

A total of 234 mothers who delivered premature infants participated in this cross-sectional study. From the third to the seventh day of the infants’ admission to the NICU, respondents completed a self-administered questionnaire to obtain data on maternal demographics, infant characteristics, obstetric and breastfeeding history, and the timing of breast milk expression initiation. In addition, mothers’ knowledge of breast milk feeding to premature infants and barriers to breast milk expression during NICU admission were assessed using a validated questionnaire. Descriptive statistics and logistic regression analyses were conducted.

**Results:**

Most mothers (87.6%) initiated breast milk expression more than six hours after delivery. Common reasons given included caesarean delivery (36.6%), maternal health problems post-delivery (24.9%), perceived insufficient milk supply (22%), and unpreparedness for premature birth (18.5%). Two factors were found to be significantly associated with delayed breast milk expression initiation: not performing skin-to-skin contact (SCC) after delivery (adjusted odds ratio (AOR) 2.60; 95% confidence interval (CI) [1.12–5.95]; *P* = 0.027), and unclear knowledge regarding the recommended frequency of milk expression when separated from the infant (AOR 15.15; 95% CI [1.97–116.76]; *P* = 0.009).

**Conclusion:**

A high proportion of mothers did not initiate breast milk expression within six hours after premature delivery. Strengthening healthcare provider support is essential in promoting early initiation of breast milk expression, emphasizing the benefits of SCC, and providing timely emotional and technical support to improve breastfeeding outcomes among premature infants.

## Introduction

Premature birth is the leading cause of neonatal mortality and is associated with long-term physical, neurodevelopmental, and socioeconomic impacts. Globally, it is estimated that 13⋅4 million babies were born prematurely (before 37 completed weeks of gestation) in 2020, a slight decrease from 13⋅8 million in 2010. It was reported that, between 2010 and 2020, approximately 15% of all premature births occurred before 32 weeks of gestation, necessitating more intensive neonatal care (Ohuma et al., 2023). In Malaysia, the premature birth rate was reported at 6.63% in 2020, with higher prevalence among Orang Asli and Indian women, and women aged over 40 (Jeganathan & Karalasingam, 2021).

A review article on optimizing nutrition in preterm low birth infants reported that premature infants are at increased risk of growth and developmental impairments compared to those born at term. As such, optimal nutrition is essential for their growth and development. Expressed breast milk (EBM) is recommended as the first feeding option due to its positive effects on cardiovascular, neurological, bone health and overall growth outcomes. Donor-pasteurised human milk is considered the second-best alternative (Kumar et al., 2017).

Initiating breastfeeding in premature infants is often more challenging due to their immaturity. This is because premature infants often exhibit reduced alertness, stamina, and coordination, making it difficult to latch, suck, and swallow (Boies & Vaucher, 2016). Consequently, stage II lactogenesis (onset of copious milk production) should begin with milk expression, as direct breastfeeding is often not feasible due to the infant’s medical condition (Nyqvist et al., 2013). A qualitative study conducted in Uganda reported that the immediate admission of premature infants to the neonatal intensive care unit (NICU) results in physical separation from the mother, further necessitating the use of EBM (Namusoke et al., 2021). In line with this, the 2009 World Health Organisation (WHO) and United Nations Children’s Fund (UNICEF) Baby-Friendly Hospital Initiative recommends “if the baby is not able to suckle, breast milk expression should begin as soon as possible after birth, preferably within 6 hours” for successful breastfeeding (World Health Organization, 2009).

In 2020, the WHO and UNICEF published the Baby-Friendly Hospital Initiative for small, sick and preterm newborns. This guideline targets healthcare workers in primary, secondary, and tertiary facilities, as well as policymakers, with the aim of establishing a supportive system of care that prioritizes the provision of human milk to small, sick, and/or preterm infants, particularly those who are initially unable to feed directly at the breast (World Health Organization & United Nations Children’s Fund, 2020). Malaysia has implemented a 20-hour breastfeeding course recommended by the WHO and UNICEF as part of a global initiative to promote, protect, and support breastfeeding. The course equips healthcare workers with essential knowledge and skills to effectively support mothers in initiating and maintaining breastfeeding (Nurjasmine, Karimah & Hamizah, 2021).

Research has shown that early initiation of breast milk expression significantly increases milk production in mothers of very low-birth-weight (VLBW) infants, and initiation of EBM within the first hour postpartum is associated with improved lactation outcomes (Parker et al., 2015). Despite recommendations to begin breast milk expression within six hours, adherence remains suboptimal (Hirpha, Mekonnen & Fenta, 2021). A cross-sectional study conducted in Finland has found that only 36.0% of mothers of premature infants initiated expression within six hours, with a median initiation time of nine hours. Furthermore, only 33.3% expressed milk more than six times daily, with a median frequency of six times (Ikonen et al., 2018). Delayed initiation of breast milk expression is linked to a higher risk of did not of exclusive breastfeeding at discharge reported by a prospective cohort study conducted in Denmark (Maastrup et al., 2014a).

Despite the well-documented benefits of breast milk in reducing morbidity and mortality among premature infants, mothers often face significant challenges in expressing breast milk (Ikonen et al., 2018). A quasi-experimental design study that was conducted in Malaysia found the stress of having a premature infant, as the infants are often admitted to the NICU for extended periods, can be overwhelming (Ong et al., 2019). In addition, frequent travel between home and hospital may hinder mother’s adherence to milk expression routines (Sisk et al., 2010). Consequently, mothers often require external motivation to maintain milk expression. Physical exhaustion and emotional distress are common (Li et al., 2024). A cross-sectional study conducted in Italy reported that 30% of mothers of premature infants experienced difficulties with milk expression, increasing the likelihood of formula feeding at discharge (Gianni et al., 2018).

This study aimed to determine the prevalence of delayed initiation of breast milk expression (beyond six hours post-delivery) and to identify factors associated with this delay among mothers of premature infants in Kelantan, Malaysia. Understanding these barriers is crucial for healthcare providers and policymakers to develop targeted strategies, interventions, and support systems during pregnancy and after premature delivery.

## Methods

### Population and sample

A cross-sectional study was conducted among mothers who delivered premature infants at two tertiary hospitals in Kelantan, Malaysia. It was conducted from January 2022 to December 2022. Mothers who had delivered singleton premature infants between 28 and 36 weeks of gestation, with the infants admitted to the NICU immediately after birth, were included in this study. Mothers were excluded if they had been diagnosed with psychiatric disorders, had infants with congenital anomalies, or had delivered extremely low birth weight infants of less than one kilogram.

A convenience sampling method was applied in this study. The sample size was calculated using a single proportion formula with a 95% confidence interval (CI). Based on a previous study in Finland, where 64% of mothers did not initiate breast expression within six hours of premature delivery (Ikonen et al., 2018). A sample size of 180 was determined with a precision of 0.07. Considering the available resources and an anticipated 30% non-response rate, a final sample size of 234 was determined for this study.

### Ethical consideration

Ethical approval was obtained from the Human Research Ethics Committee of Universiti Sains Malaysia (USM/JEPeM/20120677) on 18 April 2021 and the Ministry of Health Malaysia Medical Research and Ethics Committee (NMRR-20-2817-57640 [IIR]) on 18 May 2021. Potential respondents were informed about the study objectives and procedures, and written informed consent was obtained. Confidentiality and anonymity were strictly maintained throughout the study.

### Data collection

Data were collected using a self-administered questionnaire distributed by the main author and three trained research assistants. Mothers visiting their infants between days 3 and 7 of NICU admission were approached. Prior to approaching potential respondents, the inclusion and exclusion criteria of the participants were verified. Upon consent, respondents were guided on how to complete the questionnaire. Infant-related data were extracted from hospital records.

### Research tools

A structured questionnaire was used to collect the variables of interest. The questionnaire consisted of six sections. The first section obtained demographic information about the mothers (age, ethnicities, education level and employment status). The second section focused on infant-related data (gestational age at birth, sex, birth weight, and mode of delivery) and whether skin-to-skin contact (SSC) had occurred within the last 24 h prior to data collection. In this study, SSC was defined as placing the undressed newborn in a prone position on the mother’s bare chest, between breasts, covered with a blanket, for at least one hour (Safari et al., 2018).

The third section assessed the mothers’ obstetric and breastfeeding history, including parity, previous experiences with premature delivery, feeding methods used for their last child during the first six months, and previous experience with breast milk expression using hand, manual breast pump, or electric breast pump. The fourth section assessed current breast milk expression practices, including the timing of breast milk expression initiation after delivery (within 6 h or beyond 6 h after delivery), method and frequency of breast milk expression, reasons for delayed initiation (if applicable), and the infant’s current feeding method in the NICU.

The fifth section evaluated mothers’ knowledge regarding breast milk feeding for premature infants using a 20-item questionnaire developed and validated by Wan Nor et al. (2023). The questionnaire demonstrated good internal consistency (Cronbach’s alpha = 0.726) (Wan Nor et al., 2023). Three domains were assessed: general breastfeeding knowledge for premature infants (six items), knowledge of breast milk expression and transportation to the NICU (10 items), and knowledge of breast milk storage for ill infants (four items). Each item offered three response options, ‘true’, ‘false’, and ‘don’t know’, with correct answers scored as 1 and incorrect or ‘don’t know’ responses scored as 0. The final section of the questionnaire assessed barriers to breast milk expression using a 16-item binary response questionnaire (yes/no), also developed and validated by Wan Nor et al. (2023), with a Cronbach’s alpha of 0.736.

### Statistical analysis

All data were entered and analysed using IBM SPSS Statistics Version 28.0 software. Prior to analysis, data were checked and cleaned to ensure accuracy and completeness. Descriptive statistics were used to summarise maternal and infant characteristics, SSC practices, obstetric and breastfeeding history, timing of breast milk expression initiation, knowledge on breast milk feeding, and reported barriers to breast milk expression.

To identify factors associated with delayed initiation of breast milk expression (defined as initiation more than six hours after delivery), simple logistic regression was first conducted to screen potential predictors. Variables with a *P*-value less than 0.05 were then included in a multiple logistic regression (MLR) model. The dependent variable was binary (yes/no) based on whether breast milk expression was initiated after six hours. The independent variables included maternal socio-demographic characteristics, infant information, SSC, obstetric and breastfeeding history, knowledge of breast milk feeding, and reported barriers. Interaction terms were checked using the likelihood ratio (LR) test. Multicollinearity problems were identified using the variance inflation factor (VIF). Model fit was assessed using the Hosmer–Lemeshow goodness-of-fit test, while the model’s predictive performance was evaluated using the classification table (sensitivity and specificity) and the area under the receiver operating characteristic curve (ROC). A *P*-value of less than 0.05 was considered statistically significant.

## Results

A total of 234 mothers were invited to participate in this study, and all responded, resulting in a 100% response rate. The sociodemographic characteristics of the respondents are shown in Table 1. The mean age of the mothers was 30.6 years (SD = 5.72), while the mean age of their husbands was 33.9 years (SD = 7.67). Most of the respondents (*n* = 233 (99.6%)) were of Malay ethnicity. In terms of education, nearly half (*n* = 112 (47.9%)) had completed secondary education, while 119 respondents (49.1%) had attained tertiary education. More than half of the respondents (*n* = 130 (55.6%)) were unemployed

**Table 1:** Socio-demographic characteristics of the respondents (*n*= 234).

**Variable**	**Frequency (%)**	**Mean (SD)**
Age group (years)		
≤20	10 (4.3)	30.6 (5.72)
21–35	173 (73.9)	
>35	51 (21.8)	
Ethnicities		
Malay	233 (99.6)	
Chinese	1 (0.4)	
Highest education level		
No formal education/primary	7 (3.0)	
Secondary	112 (47.9)	
Tertiary	115 (49.1)	
Employment status		
Employed	104 (44.4)	
Unemployed	130 (55.6)	
Marital Status		
Married	234(100%)	
Divorce/single	0 (0%)	

Table 2 shows information on the infants and the practice of SSC between mothers and their infants within the 24 h preceding data collection. Most of the infants (*n* = 184 (78.6%)) were born between 33 and 36 weeks of gestation. More than half (*n* = 125 (53.4%)) were boys, and 156 (66.7%) had a low birth weight ranging from 1,500 to 2,499 grams. Additionally, 137 (58.5%) of the infants were delivered *via* caesarean section. Regarding SSC, more than half of the mothers (*n* = 134 (58.5%)) did not engage in SSC during the 24 h prior to data collection. Among those who did, more than half (*n* = 63 (65%)) reported SSC sessions lasting less than one hour.

**Table 2:** Infant information and the practice of skin-to-skin contact between mothers and infants.

**Variable**	**Frequency (%)**
Gestational age at delivery (weeks)	
28–32 (very premature)	50 (21.4)
33–36 (moderately premature)	184 (78.6)
Sex	
Boy	125 (53.4)
Girl	109 (46.6)
Birth weight (grams)	
<1,500 (very low birth weight)	28 (12.0)
1,500–2,499 (low birth weight)	156 (66.7)
≥2,500 (normal)	50 (21.4)
Method of delivery	
Vaginal	97 (41.5)
Caesarean section	137 (58.5)
SSC in the last 24 h	
Yes	97 (41.5)
No	134 (58.5)
Duration of SSC (*n*= 97)	
<1 h	63 (65.0)
1 h	17 (17.5)
>1 h	17 (17.5)

Table 3 shows the respondents’ previous obstetric and breastfeeding profiles. Most mothers (*n* = 142 (60.7%)) were multiparous. About one-third (*n* = 49 (34.5%)) reported a history of previous premature delivery. In terms of infant feeding practices, more than half of the respondents (*n* = 87 (61.3%)) had prior experience of exclusively breastfeeding their last child for the first six months. Additionally, 79 mothers (53.5%) used an electric breast pump to express their breast milk.

**Table 3:** Previous obstetric and breastfeeding profile.

**Variable**	**Frequency (%)**
Parity	
Para 1	92 (39.3)
Para ≥ 2	142 (60.7)
Previous premature delivery (*n*= 142)	
Yes	49 (34.5)
No	93 (65.5)
Number of previous premature infants (*n*= 49)	
1 child	24 (49.0)
2 children	19 (38.8)
3 children and above	6 (12.2)
Last child feeding method for the first six months (*n* =142)	
Breast milk only	87 (61.3)
Breast milk and other types of drinks at certain time only	6 (4.2)
Formula milk besides breast milk	48 (33.8)
Formula milk only	1 (0.7)
Method of breast milk expression (*n* =142)^*^	
Hands	48 (33.8)
Manual breast pump	50 (35.2)
Electric breast pump	76 (53.5)

**Notes.**

* Respondents can choose more than one method.

Table 4 shows the mothers’ experiences regarding the timing of breast milk expression initiation and the feeding methods used while their babies were in the NICU. Most respondents (*n* = 205 (87.6%)) initiated breast milk expression more than six hours after the delivery of premature infants. Common reasons given for delayed initiation included having undergone a caesarean section (*n* = 75 (36.6%)), experiencing maternal health problems post-delivery (*n* = 51 (24.9%)), perceiving an inadequate breast milk supply (*n* = 45 (22.0%)), and feeling unprepared for premature delivery (*n* = 38 (18.5%)). Among the 29 (12.4%) mothers who initiated breast milk expression within six hours after delivery, nearly half (*n* = 13 (44.8%)) used an electric breast pump, with most (*n* = 21 (72.4%)) expressing milk four times or fewer per day. Regarding infant feeding practices in the NICU, (*n* = 99 (42.3%)) of the infants received breast milk exclusively during their hospital stay in the NICU.

**Table 4:** Timing of breast milk expression initiation and infant feeding methods in the NICU after delivery of premature infants.

**Variable**	**Frequency (%)**
Timing of breast milk expression initiation after delivery of premature infants	
≤6 h	29 (12.4)
>6 h	205 (87.6)
Methods of breast milk expression (*n* =29)^*^	
Hands	12 (41.4)
Manual breast pump	9 (31.0)
Electric breast pump	13 (44.8)
Frequency of breast milk expression in the last 24 h (*n*= 29)	
≤4 times	21 (72.4)
5–8 times	8 (27.6)
>8 times	0 (0.0)
Reasons for delayed breast milk expression (*n* =205)^*^	
Unprepared for premature delivery	38 (18.5)
Maternal health problems post delivery	51 (24.9)
Infant health problems after birth	41 (20.0)
Perceived an inadequate milk supply	45 (22.0)
Caesareans delivery	75 (36.6)
Infant feeding method in the NICU	
Breast milk only	99 (42.3)
Formula milk, besides breast milk	123 (52.6)
Not yet started (infants are nil by mouth)	12 (5.1)

**Notes.**

* Respondents can choose more than one method.

Table 5 shows the distribution of correct and incorrect or ‘don’t know’ responses for each item assessing knowledge regarding breast milk feeding to premature infants. A large majority of the respondents knew that premature infants should receive breast milk (*n* = 224 (95.7%)), and breast milk reduces the risks of necrotising enterocolitis (*n* = 197 (84.2%)) and meningitis (*n* = 190 (81.2%)). However, 172 (72.2%) of respondents were unclear about whether premature infants did not require special formula milk when expressed breast milk is available.

**Table 5:** Knowledge regarding breast milk feeding to premature infants.

**Variable**	**Frequency (%)**	
	**Correct response**	**Incorrect/Don’t know response**
a) General breastfeeding knowledge for premature infants		
Premature infants should receive breast milk	224 (95.7)	10 (4.3)
Premature infants require special formula milk, though expressed breast milk was prepared for the infant	65 (27.8)	169 (72.2)
Premature infants should receive other drinks other than breast milk, such as plain water	187 (79.9)	47 (20.1)
Premature infants should receive exclusive breast milk for the first 6 months	161 (68.8)	73 (31.2)
Breast milk reduces the risk of necrotizing enterocolitis in preterm infants	197 (84.2)	37 (15.8)
Breast milk reduces the risk of meningitis in preterm infants	190 (81.2)	44 (18.8)
b) Knowledge regarding breastmilk expression and transportation to NICU		
Mothers who are not with their infants should start breast milk expression within the first six hours after birth	158 (67.5)	76 (32.5)
Breast milk expression needs to be done every 3 h if the premature baby is away from the mother	167 (71.4)	67 (28.6)
Moist heating and massage before breast milk expression can promote milk secretion	204 (87.2)	30 (12.8)
Expressed breast milk needs clear labelling of name of infant, date, and time of expression.	223 (95.3)	11 (4.7)
Breast milk expression can be done simultaneously on both sides of the breast	184 (78.6)	50 (21.4)
Expressed breast milk may be mixed with previously expressed milk	184 (78.6)	50 (21.4)
The leftover expressed breast milk that has been used may be stored again	144 (61.5)	90 (38.5)
Expressed breast milk may be warmed on a fire	174 (74.4)	60 (25.6)
Expressed breast milk may be warmed in a microwave	137 (58.5)	97 (41.5)
Expressed breast milk transported to the hospital must be kept chilled in a cool box with ice or cooler pack	206 (88.0)	28 (12.0)
c) Knowledge regarding breastmilk storage for ill infants		
Expressed breast milk may be stored for 4 h in room temperature	149 (63.7)	85 (36.3)
Expressed breast milk may be stored for 48 in a lower part of a refrigerator	122 (52.1)	112 (47.9)
Expressed breast milk may be stored for 2 weeks in a freezer of a 1-door refrigerator	139 (59.4)	95 (40.6)
Expressed breast milk may be stored for 3 months in a freezer of a 2-door refrigerator	140 (59.8)	94 (40.2)

Regarding knowledge related to breast milk expression and transportation to the NICU, about one-third of the respondents (*n* = 76 (32.5%)) were unclear about whether mothers who are separated from their infants should initiate breast milk expression within the first six hours after birth. Similarly, 67 (28.6%) respondents were unclear about the frequency of breast milk expression, which may be done every 3 h when the baby is not with the mother, and 90 (38.5%) respondents were unclear about the fact that leftover expressed breast milk that has been used cannot be stored again. Most respondents knew that moist heat and massage before breast milk expression can promote milk secretion (*n* = 204 (87.2%)), and expressed breast milk must be clearly labelled with the infant’s name, date, and time of expression (*n* = 223 (95.3%)), and that it should be transported to the hospital in a cool box with ice or cooler pack (*n* = 206 (88.0%)).

In the domain of breast milk storage for ill infants, nearly half of the respondents answered were unsure about the appropriate storage durations: (*n* = 112 (47.9%)) for storing expressed breast milk in the lower part of a refrigerator for 48 h, (*n* = 95 (40.6%)) for storing it in the freezer of a one-door refrigerator for two weeks, (*n* = 94 (40.2%)) for storing it in the freezer of a two-door refrigerator for up to three months.

Table 6 shows the barriers to breast milk expression among mothers with infants admitted to the NICU. Most of the respondents (*n* = 228 (97.4%)) did not feel embarrassed using a breast pump to express breast milk, 229 (97.9%) respondents reported having supportive husbands and families, and 230 (98.3%) respondents felt supported by hospital staff in their efforts to express breast milk. However, nearly one-quarter of the mothers indicated that physical exhaustion (*n* = 52 (22.2%)) and concerns about inadequate milk supply (*n* = 55 (23.5%)) interfered with their ability to express breast milk.

**Table 6:** Barriers to breastmilk expression while infants are in the NICU.

**Variable**	**Frequency (%)**	
	**Yes**	**No**
I have a problem expressing breast milk because I am not prepared for premature delivery	36 (15.4)	198 (84.6)
Feelings of stress with premature delivery hindered me from expressing breast milk	23 (9.8)	211 (90.2)
I feel uncomfortable while expressing breast milk	26 (11.1)	208 (88.9)
I experience a lack of privacy while expressing milk	24 (10.3)	210 (89.7)
I feel embarrassed to express milk using a breast pump	6 (2.6)	228 (97.4)
I feel the act of breast milk expression is tiring	17 (7.3)	217 (92.7)
My body feels too tired to express breast milk	52 (22.2)	182 (77.8)
My husband is less supportive of me regarding breast milk expression	5 (2.1)	229 (97.9)
The family is less supportive of me regarding breast milk expression	5 (2.1)	229 (97.9)
I felt the hospital staff was less supportive of me about breast milk expression	4 (1.7)	230 (98.3)
I don’t have enough time to express breast milk	14 (6.0)	220 (94.0)
I feel that breast milk expression is a waste of time.	3 (1.3)	231 (98.7)
Expressing breast milk causes pain in my breasts	33 (14.1)	201 (85.9)
Inadequate milk supply interrupted my breast milk expression activity	55 (23.5)	179 (76.5)
I do not have a breast pump at home	16 (6.8)	328 (93.2)
The difficulty in delivering the expressed milk from home to the hospital discourages me from expressing breast milk	10 (4.3)	224 (95.7)

Table 7 presents the results of the simple and multiple logistic regression analyses conducted to identify factors associated with delayed initiation of breast milk expression. In the simple logistic regression analysis, two variables were found to have *P*-values less than 0.05 and were subsequently included in the multiple logistic regression analysis. These variables were a practice of SSC and knowledge regarding the recommended frequency of breast milk expression when separated from infants. Both variables remained significantly associated with delayed initiation in the final model. Mothers who did not perform SSC after delivery had 2.6 times higher odds of delaying breast milk expression beyond six hours post-delivery of premature infants compared to those who engaged in SSC. Additionally, mothers who were unclear that breast milk expression may be done every 3 h when separated from their premature infant had 15.2 times higher odds of delayed initiation compared to those who answered correctly.

**Table 7:** Factors associated with delayed initiation of breast milk expression.

**Variables**	**Crude OR** ^a^ **(95% CI)**	**Adjusted OR** ^b^ **(95% CI)**	**Wald** ^b^ **Statistic (df)**	**p-value** ^b^
SSC in the last 24 hours				
Yes	1	1		
No	2.6 (1.17, 5.82)	2.6 (1.12, 5.95)	4.91 (1)	0.027
Breast milk expression may be done every 3 h if a premature baby is away from the mother				
Correct	1	1		
Incorrect/don’t know	13.3 (1.77, 99.83)	15.2 (1.97, 116.76)	6.81 (1)	0.009

**Notes.**

a Simple Logistic regression

b Multiple logistic regression

OR, Odds Ratio.

CI, Confidence interval.

df, degree of freedom.

The Hosmer–Lemeshow goodness of fit test *p*-value = 0.916.

The percentage of correct classification is 87.6%.

The area under ROC curve = 0.739.

There are no interaction and multicollinearity problems.

No significant interaction terms were detected, and multicollinearity was not a concern as all VIF values were below 10. The model demonstrated a good fit, as indicated by a non-significant Hosmer–Lemeshow goodness of fit test (*P* = 0.916). The classification table showed an overall accuracy of 87.6%, exceeding the 70% threshold typically considered indicative of a good model. Additionally, the area under the receiver operating characteristic (ROC) curve was 0.739, suggesting that the model could correctly discriminate 73.9% of the cases.

## Discussion

In this study, most respondents (87.6%) initiated breast milk expression more than six hours after delivery. Our finding is consistent with a cross-sectional study conducted in Italy, which reported a similar rate of 88% (Gianni et al., 2018), but lower than the 100% reported in a cross-sectional study from Ethiopia (Hirpha, Mekonnen & Fenta, 2021). However, the rate observed in our study was higher compared to findings from Finland (64.0%), Denmark (77.0%), and Germany (61.8%) (Ikonen et al., 2018; Maastrup et al., 2014b; Scholten et al., 2022). A study conducted in Florida highlighted the importance of early initiation of milk expression, showing that mothers who began milk expression within one hour of delivery produced significantly more breast milk at six weeks postpartum compared to those who started later (Parker et al., 2015). Furthermore, a prospective cohort study in Denmark found that delayed initiation of breast milk expression beyond 48 h postpartum has been associated with a higher risk of did not to exclusively breastfeeding at discharge (Maastrup et al., 2014b)). The International Lactation Consultant Association recommends that early stimulation or breast milk expression (within 6 h) and frequent stimulation are associated with higher milk production later on(Nyqvist et al., 2013).

These findings underscore the critical role of timely support and counselling from health care providers, including nurses and doctors, lactation consultants and counsellors in encouraging early breast milk expression, ideally within the first six hours postpartum, even while mothers and infants are still in the labour room awaiting NICU admission (Laborie et al., 2020; Shattnawi, 2017). Hospital-based nurses and midwives play a pivotal role in promoting, protecting, and supporting breastfeeding (Chen & Chen, 2025). It is essential for them to thoroughly understand their roles in breastfeeding support for mothers of preterm infants and to acquire comprehensive knowledge of appropriate care for milk expression to optimise breastfeeding outcomes. Evidence shows that education programs enhance nurses’ and midwives’ knowledge and attitudes toward supporting mothers of preterm infants in expressing breast milk (Tanaka & Horiuchi, 2021). In Malaysia, the existing 20-hour breastfeeding course for healthcare providers (Nurjasmine, Karimah & Hamizah, 2021) could be strengthened by placing greater emphasis on early initiation of milk expression within 6 h and strategies to maintain lactation following premature delivery.

In addition, a comprehensive antenatal and postnatal program is necessary to prepare mothers of preterm infants for breast milk expression, including techniques, storage, transportation, and maintaining milk quality. This empowers mothers to continue breastfeeding and transition to direct breastfeeding when possible (Seah & Cheah, 2017). In Malaysia, targeted antenatal education for mothers at risk of premature delivery is currently practised in some hospitals, though implementation is not yet universal. This education covers the timing of breast milk expression, methods of expression (manual or pump), and proper storage and transport of expressed milk to the NICU. However, because many mothers do not anticipate preterm birth, antenatal education alone is insufficient. A structured follow-up program is needed to offer immediate postpartum support, including clear protocols, ongoing counselling, and technical assistance. Developing a comprehensive antenatal and postnatal module may help address barriers to timely expression and improve the availability of mothers’ own milk for premature infants, ultimately enhancing breastfeeding rates in this vulnerable population.

Apart from that, mothers of premature infants often experience routine separation immediately after birth, and they need to deal with milk expression with prolonged separation from their infants (Namusoke et al., 2021). Therefore, opportunities should be provided for mothers and preterm infants to remain together, with mothers having 24-hour access to their infants after preterm delivery. Evidence highlights the importance of maternal interaction in caring for premature infants, as it fosters bonding and attachment, both of which are crucial for healthy child development. Healthcare professionals must therefore create supportive care environments that prioritize the protection and safety needs of both infants and mothers (Joaquim et al., 2018).

Among the reasons given by our respondents for delayed initiation, caesarean delivery was the most common (36.6%). This aligns with findings from Finland, where caesarean section was identified as a significant predictor of delayed breast milk expression (Ikonen et al., 2018). In this study, 58.5% of respondents underwent caesarean delivery. Previous research has consistently shown that caesarean birth is associated with lower rates of early breastfeeding initiation (Johar et al., 2020; Mary et al., 2021). A prospective cohort study conducted in Malaysia reported that the reasons could be due to postoperative pain, the effects of anaesthesia, and delayed maternal-infant bonding. Therefore, it is essential for healthcare providers to offer targeted support to mothers who deliver *via* caesarean section, particularly those with premature births (Johar et al., 2020). Antenatal education should also emphasize the importance of early breast milk expression and prepare mothers for potential challenges following surgical delivery (Johar et al., 2020). Interestingly, despite caesarean delivery being identified as the main barrier to early initiation, approximately 20% of mothers who underwent a caesarean section did not perceive it as such, possibly due to postoperative support and timely assistance provided by healthcare staff in initiating breastfeeding (Khan, Verma & Gupta, 2024).

Other reported reasons for delayed initiation included concerns about inadequate breast milk supply (22%) and feeling unprepared for premature birth (18.5%). These findings are supported by previous cross-sectional and qualitative studies , which found that maternal anxiety about inadequate milk supply and lack of readiness for premature delivery often hinders frequent milk expression, increasing the likelihood of formula feeding at discharge (Gianni et al., 2018; Sisk et al., 2010). A systematic review reported that perceived insufficient milk supply affects 10% to 25% of mothers and is a leading cause of early breastfeeding cessation, with approximately half of mothers discontinuing breastfeeding at various postpartum stages due to this concern (Huang et al., 2022). Thus, it is crucial for hospital staff to provide reassurance and education to help mothers distinguish between perceived and actual milk insufficiency. Empowering mothers with knowledge and support can significantly improve breastfeeding outcomes (Westerfield, Koenig & Oh, 2018).

Our study found a strong association between the initiation of breast milk expression and the implementation of SSC between mothers and their premature infants. Mothers who did not perform SSC had 2.6 times higher odds of delaying breast milk expression beyond six hours postpartum compared to those who did. The findings of the current study are in agreement with a quasi-experimental study conducted in Iraq, which reported a significant association between SSC and the timely initiation of breast milk expression (Safari et al., 2018). Similarly, a cross-sectional study in sub-Saharan Africa found that mothers who practised SSC were more likely to initiate breastfeeding early compared to those who did not (Aboagye et al., 2023). In China, mothers who engaged in SSC were twice as likely to exclusively breastfeed their premature infants at discharge compared with mothers who did not (Zhang et al., 2020). A randomized controlled trial study in India also demonstrated that early SSC among low-birth-weight neonates significantly improved exclusive human milk feeding both during hospitalisation and up to one month post-discharge (Jayaraman et al., 2017). In Malaysia, a longitudinal quasi-experimental study found that mothers who received structured education and close monitoring of SSC practices had higher breastfeeding rates and shorter hospital stays compared to those in the control group (Samsudin et al., 2023).

These findings highlight the critical role of early SSC in promoting exclusive breastfeeding and timely milk expression among mothers of premature infants. Despite this, our study showed that 58.5% of mothers did not perform SSC in the 24 h prior to data collection, and among those who did, more than half (65%) engaged in SSC for less than 1 h per session.

The guidelines by WHO and UNICEF suggest that small, sick and/or preterm infants should be placed in SSC care on the mother as soon after birth if the mother’s and infant’s conditions in a stable condition (World Health Organization & United Nations Children’s Fund, 2020). Another guidelines by WHO on kangaroo mother care (KMC) recommend that KMC for preterm or low-birth-weight infants should be started as soon as possible after birth, unless the infant is unable to breathe spontaneously after resuscitation, in shock, or requires mechanical ventilation (Darmstadt et al., 2023; World Health Organization, 2023). It is recommended that KMC should be given for 8 to 24 h per day or as many hours as possible (Darmstadt et al., 2023). These recommendations reaffirm the pivotal role of preventive and promotive care for preterm and low birth weight infants, particularly emphasizing the importance of keeping the mother and baby together, and empowering families to provide competent and confident care for their premature newborns (Darmstadt et al., 2023).

In our study, the lower SSC rates may be attributed to the challenges of performing SSC in NICU settings, where both parents and healthcare providers may perceive it as risky for the infants’ clinical stability (Bedetti et al., 2023). A systematic review identified maternal fears of harming their infants, physical discomfort, and fatigue as the main barriers to practising SSC. From the healthcare provider’s perspective, concerns about the infants’ condition, increased workload, and lack of training or guidelines were the main obstacles (Seidman et al., 2015). These barriers must be addressed, as a systematic review and meta-analysis reported that SSC has been shown to reduce the risks of mortality, severe infection, and sepsis in premature or low birth weight infants. Studies have confirmed the safety and feasibility of early SSC in NICU settings, with no adverse effects on the physiological stability of premature infants (Bedetti et al., 2023; Kristoffersen et al., 2023). Therefore, healthcare providers must be equipped with the knowledge and confidence to encourage SSC, given its short- and long-term benefits for premature infants (Bedetti et al., 2023).

Our study found that mothers who were unclear about breast milk expression may be done every three hours when separated from their infants had 15.2 times higher odds of delaying initiation beyond six hours. Notably, 28.6% of mothers were unaware of this recommended frequency. Among the 12.4% of mothers who did initiate breast milk expression within six hours, 72.4% expressed milk 4 times or fewer per day. Knowledge regarding breast milk expression frequency is very important, as the WHO and UNICEF Baby-Friendly Hospital Initiative (2009) recommend expressing breast milk at least six times in 24 h, for approximately 20 min each session, when direct breastfeeding is not possible (World Health Organization, 2009). Supporting this, a study in Germany found that 45.9% of premature infants received exclusive maternal milk when mothers expressed 7-9 times daily in the first three days, compared to 26.3% whose mothers expressed 4-6 times daily, and only 15.4% when expression occurred 1-3 times daily (Scholten et al., 2022). Similarly, an observational prospective study in China reported that mothers who expressed milk six or more times per day successfully achieved full lactation for their premature infants and produced foremilk with higher levels of fat, carbohydrates, and energy by day seven (Ru, Huang & Feng, 2020). These findings highlight the need to educate and support mothers, especially those separated from their infants in the NICU, on the importance of expressing breast milk more than six times daily to establish an adequate milk supply and reduce risks associated with delayed breastfeeding (Li et al., 2024; World Health Organization, 2009).

This study has several limitations. First, as a cross-sectional study, it cannot establish causality since data were collected between days 3 and 7 postpartum, and it is subject to temporal ambiguity, recall bias, and selection bias. Second, there is limited information on healthcare provider training and practices in supporting mothers of premature infants in initiating breast milk expression in facilities. This gap may contribute to delays in initiating breast milk expression. Third, the findings may not be generalisable to all mothers of premature infants in Malaysia, as the sample was limited to two tertiary hospitals in Kelantan and employed convenience sampling. Additionally, the study population was predominantly Malay, which may limit the applicability of the results to more ethnically diverse populations. Future research should consider a larger, more representative sample across multiple regions and ethnic groups to enhance generalisability and provide a more comprehensive understanding of the factors influencing breast milk expression among mothers of premature infants.

## Conclusion

This study found a high proportion of mothers-initiated breast milk expression more than six hours after premature delivery. Two factors were significantly associated with delayed initiation: the absence of SSC and limited maternal knowledge regarding the recommended frequency of breast milk expression when separated from their infant. These findings highlight the critical importance of strengthening healthcare provider training to ensure routine implementation of practices that minimize mother–infant separation, enable immediate and sustained SSC, and support early (within six hours) and frequent milk expression for mothers of preterm infants. In addition, hospitals should establish monitoring and evaluation mechanisms, such as audit tools, checklists, and periodic competency assessments, to verify that training translates into consistent bedside practice across labour wards, postnatal units, and NICUs. Additionally, comprehensive lactation education should be integrated into maternal care, beginning in the antenatal period and extending through the immediate postpartum phase. Special attention should be directed toward mothers at risk of preterm birth, ensuring that they receive timely guidance and practical support to initiate milk expression as soon as possible after delivery. Emphasis should also be placed on the benefits of early SSC and the importance of beginning breast milk expression within the first six hours postpartum.

## Supplemental Information

10.7717/peerj.21159/supp-1 Supplemental Information 1 Data

10.7717/peerj.21159/supp-2 Supplemental Information 2 STROBE checklist

10.7717/peerj.21159/supp-3 Supplemental Information 3 Questionnaire

## Additional Information and Declarations

### Data Availability

The following information was supplied regarding data availability: The raw data is available in the Supplemental File.
