# Supplementary material for: Determinants of delayed initiation of breast milk expression among mothers of premature infants in Kelantan, Malaysia
Source: PeerJ. 2026 Apr 23;14:e21159. doi: 10.7717/peerj.21159 (PMC13110654; doi:10.7717/peerj.21159)
Supplement: Supplemental Information 3 [file peerj-14-21159-s003.docx]

# SOALAN KAJI SELIDIK

**TAJUK KAJIAN:**

**FAKTOR BERKAITAN DENGAN IBU TIDAK MENGAMALKAN PERMULAAN PEMERAHAN SUSU IBU DAN PEMBERIAN SUSU IBU SECARA EKSKLUSIF SELEPAS KELAHIRAN BAYI PRAMATANG DI KELANTAN**

**BAHAGIAN 1: UNTUK DI ISI OLEH IBU PADA HARI KE-3 HINGGA KE- 7 SEMASA BAYI BERADA DI WAD)**

**SEKSYEN A: MAKLUMAT RESPONDEN**

1. **Data Sosiodemografi**
2. Nama:
3. No Kad Pengenalan:
4. RN:
5. Bayi dimasukkan di wad mana:

1 Nilam

2 Nilam

1. No telefon 1:___________________
2. No telefon 2: ___________________
3. Umur anda : _____________ (tahun)
4. Umur Suami :______________ (tahun)
5. Bangsa

Melayu

Cina

India

Lain-lain

Nyatakan: ___________________

1. Taraf pendidikan

| Tiada pendidikan formal |  | Diploma |  |
| --- | --- | --- | --- |
| Sekolah rendah |  | Ijazah |  |
| PMR/SRP/PT3 |  | Ijazah lanjutan |  |
| SPM/STPM |  |  |  |

1. Adakah anda bekerja?

| Ya | Tidak |
| --- | --- |

Jika YA, nyatakan jenis pekerjaan: ______________________

6. Pekerjaan suami: _______________________

7. Anggaran pendapatan keluarga (sebulan): RM ___________________

8. Anggaran jarak antara rumah anda dengan hospital ini: _______________(km)

9.Alamat Anda: ________________________________________________________

________________________________________________________

10. Bilangan anak yang dilahirkan hidup: ________________ orang

11. Berapakah jarak kelahiran dengan anak yang terakhir? ________________Bulan

________________Tahun

12. Adakah anda pernah melahirkan bayi pra-matang sebelum ini: Ya Tidak

Jika ya, berapakah jumlah anak yang dilahirkan pramatang? __________orang

13. Adakah anak pramatang sebelum ini pernah dimasukkan ke wad bayi?

Ya Tidak

1. **Maklumat Penyusuan Sebelum ini**

**(Abaikan soalan 1-2 pada bahagian ini, jika ini adalah kelahiran sulong)**

1. Apakah kaedah penyusuan bayi dalam enam bulan pertama bagi bayi terakhir sebelum kelahiran ini? (Pilih satu dan tandakan √)

| 1. Memberi susu ibu sahaja |  |
| --- | --- |
| 1. Memberi susu ibu, air masak atau minuman lain pada masa-masa tertentu sahaja |  |
| 1. Memberi susu formula di samping susu ibu |  |
| 1. Memberi susu formula sahaja |  |

(WHO, 1991)

1. Jika anda mempunyai pengalaman memberi susu perahan ibu, apakah kaedah yang digunakan untuk memerah susu sebelum ini? (Boleh pilih lebih daripada satu dan tandakan √)
2. Memerah susu menggunakan tangan
3. Pam susu manual
4. Pam susu eletrik
5. Lain-lain

**(c). Maklumat penyusuan sekarang**

1. Adakah anda menerima nasihat mengenai penyusuan susu ibu daripada anggota kesihatan semasa mengandung pada kali ini?

Ya Tidak

1. Berapa lama anda merancang untuk menyusukan bayi dengan memberi susu ibu sahaja?

________________bulan/tahun

1. Kaedah penyusuan yang dirancang selama enam bulan pertama kelahiran bayi anda. (Pilih satu dan tandakan √)

| 1. Memberi susu ibu sahaja |  |
| --- | --- |
| 1. Memberi susu ibu, air masak atau minuman lain pada masa-masa tertentu sahaja |  |
| 1. Memberi susu formula di samping susu ibu |  |
| 1. Memberi susu formula sahaja |  |

(WHO,1991)

1. Apakah yang diberikan kepada bayi anda semasa berada di wad bayi (Pilih satu dan tandakan √)

| 1. Memberi susu ibu sahaja |  |
| --- | --- |
| 1. Memberi susu ibu, air masak atau minuman lain pada masa-masa tertentu sahaja |  |
| 1. Memberi susu formula di samping susu ibu |  |
| 1. Memberi susu formula sahaja |  |
| 1. Belum dibenarkan menerima susu ibu atau susu formula (dipuasakan) |  |

1. Bilakah anda mula memerah susu selepas kelahiran? (Pilih **satu** dan tandakan √)

| 1. Dalam tempoh 6 jam selepas kelahiran |  |
| --- | --- |
| 1. Lebih dari 6 jam selepas kelahiran |  |
| 1. Belum mula memerah susu |  |

**Jika anda belum memulakan pemerahan susu, teruskan dengan soalan 7 sahaja bagi bahagian (c ) ini*

1. Nyatakan masa (anggaran )sebenar anda memulakan pemerahan susu:

__________jam selepas kelahiran

__________ hari selepas kelahiran

1. Jika anda memulakan perahan selepas 6 jam atau belum memulakan perahan, Nyatakan sebab- sebabnya: (Boleh pilih lebih daripada satu pilihan dan tandakan √)

| 1. Tidak bersedia dengan kelahiran pramatang |  |
| --- | --- |
| 1. Masalah kesihatan ibu selepas kelahiran |  |
| 1. Masalah kesihatan bayi selepas kelahiran |  |
| 1. Merasakan susu ibu tidak cukup untuk bayi pramatang |  |
| 1. Kelahiran secara pembedahan |  |
| 1. Sebab-sebab lain (Nyatakan):   1.______________________________  2.______________________________  3.______________________________ |  |

(Lui *et al.,* 2013 & Morag *et al.,* 2016)

1. Kaedah yang digunakan untuk memerah susu (Boleh pilih lebih daripada satu pilihan dan

tandakan √)

i. Memerah susu menggunakan tangan

ii. Pam susu manual

iii. Pam susu elektrik

iv Lain-lain

9. Kekerapan memerah susu dalam sehari (dalam masa 24 jam lalu) :_________(kali)

10. Adakah suami anda menggalakkan anda untuk mengamalkan penyusuan susu ibu?

Ya Tidak

11. Siapakah yang paling banyak menggalakkan anda untuk mengamalkan penyusuan susu ibu? (pilih satu sahaja dan tandakan √)

i. Suami

ii. Ahli keluarga

iii. Rakan

iv. Kakitangan kesihatan

v. Tiada

vi. Lain-lain (nyatakan): ___________________

12. Adakah susu perahan diserahkan kepada jururawat/doktor untuk diberikan kepada bayi?

Ya Tidak

1. Jika jawapan anda adalah **‘TIDAK’** bagi soalan 12, nyatakan sebab-sebab susu perahan

tidak diserahkan kepada jururawat/doktor?

|  | Kesukaran mengepam |  |
| --- | --- | --- |
|  | Tidak cukup susu |  |
|  | Jarak jauh antara rumah dan hospital |  |
|  | Tiada ahli keluarga untuk menghantar susu ke hospital |  |
|  | Kurang mendapat bimbingan/nasihat secukupnya |  |
|  | Lain-lain:   1. _________________________________ 2. _________________________________ |  |

(Alves *et al.,* 2013)

**(d). Maklumat sentuhan kulit ke kulit dan *kangaroo mother care (KMC)***

Definisi *Kanggaroo Mother Care* (KMC) adalah penjagaan bayi pramatang yang yang melibatkan sentuhan kulit ke kulit antara ibu dengan ibu. Ciri-ciri utamanya merangkumi sentuhan kulit ke kulit awal, berterusan dan berpanjangan antara ibu dan bayi. (WHO, 2015).

KMC secara asasnya adalah kedudukan seperti kanggaru di mana bayi diletakkan dan dipegang dalam sentuhan kulit-ke-kulit secara terus di dada ibu dalam kedudukan menegak (Rujuk Rajah 1). Tujuannya adalah untuk permulaan awal KMC dan untuk memastikan kesinambungan secara berterusan (lebih dari 18 jam sehari). Namun begitu, permulaan, kesinambungan dan jangka masa melakukan KMC mungkin berbeza-beza sesuai dengan kestabilan bayi dan perawatan yang diterima oleh bayi (Vesel et *al.,* 2015).

| Definisi sentuhan kulit ke kulit adalah bayi diletakkan di dada ibu tanpa pakaian yang mana dada bayi bersentuhan secara langsung dengan dada ibu dan kedua-duanya akan ditutupi selimut dan dibiarkan sekurang-kurangnya selama satu jam atau lebih (WHO & UNICEF, 2009) |
| --- |


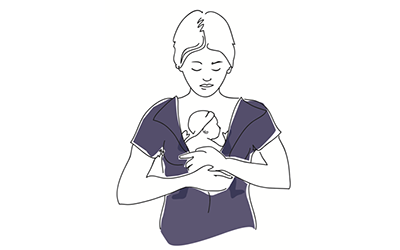


Rajah 1

**Jika anda belum melakukan sentuhan kulit ke kulit atau kangaroo mother care (KMC) , abaikan soalan di bahagian ini (Nombor1-4).*

1.Adakah anda melakukan sentuhan kulit ke kulit atau *kanggaroo mother care (KMC)* bersama bayi anda dalam tempoh 24 jam yang lalu?

Ya Tidak

Jika **YA**, teruskan dengan soalan nombor 4

2.Berapa lamakah anda melakukan sentuhan kulit ke kulit atau *kanggaroo mother care (KMC)* bersama bayi anda?

|  | Kurang 1jam |  |
| --- | --- | --- |
|  | 1 jam |  |
|  | Lebih daripada 1 jam |  |

(Maastrup *et al.,* 2014 & WHO, 2003)

**SEKSYEN B: PENGETAHUAN BERKAITAN PEMBERIAN SUSU IBU KEPADA BAYI PRAMATANG (KNOWLEDGE ON GIVING BREAST MILK TO PREMATURE INFANTS)**

**(a). Pengetahuan umum tentang penyusuan susu ibu bagi bayi pramatang**

| **No item** | **Pengetahuan umum tentang susu ibu dalam kalangan bayi pramatang** | **Ya** | **Tidak** | **Tidak**  **tahu** |
| --- | --- | --- | --- | --- |
| 1 | Bayi pramatang perlu diberikan susu ibu. |  |  |  |
| 2 | Bayi pramatang memerlukan susu formula khas pramatang di samping susu perahan ibu |  |  |  |
| 3 | Bayi pramatang memerlukan minuman lain selain  daripada susu ibu seperti air masak. |  |  |  |
| 4 | Bayi pramatang perlu diberikan susu ibu sahaja selama 6  bulan pertama |  |  |  |
| 5 | Susu ibu mengurangkan risiko radang pada usus bayi pramatang. |  |  |  |
| 6 | Susu ibu dapat mengurangkan risiko jangkitan kuman pada selaput otak bayi pramatang. |  |  |  |

(**b). Pengetahuan tentang pemerahan susu ibu dan cara susu ibu dibawa ke wad bayi**

| **No**  **Item** | **Pengetahuan tentang pemerahan susu ibu dan cara susu ibu dibawa ke unit rawatan rapi** | **Ya** | **Tidak** | **Tidak tahu** |
| --- | --- | --- | --- | --- |
| 1 | Ibu yang tidak bersama bayinya perlu memulakan pemerahan susu ibu dalam tempoh 6 jam pertama selepas kelahiran. |  |  |  |
| 2 | Ibu yang tidak bersama bayinya, perlu memerah susu setiap 3 jam |  |  |  |
| 3 | Demahan panas dan urutan sebelum pemerahan susu dapat merangsang pengeluaran susu. |  |  |  |
| 4 | Susu perahan perlu dilabel dengan nama bayi, tarikh dan masa susu itu diperah. |  |  |  |
| 5 | Perahan susu boleh dilakukan serentak pada kedua-dua belah payudara. |  |  |  |
| 6 | Susu perahan boleh dicampur dengan susu perahan sebelumnya |  |  |  |
| 7 | Lebihan susu perahan yang telah diberikan kepada bayi boleh disimpan semula. |  |  |  |
| 8 | Susu perahan boleh dipanaskan di atas api. |  |  |  |
| 9 | Susu perahan boleh dipanaskan dalam ketuhar gelombang mikro. |  |  |  |
| 10 | Susu perahan yang dibawa ke hospital mesti disimpan di dalam kotak ais yang berisi ais atau pek sejuk. |  |  |  |

**(c). Pengetahuan tentang penyimpanan susu ibu untuk bayi yang tidak sihat**

| **No**  **Item** | **Pengetahuan tentang penyimpanan susu ibu untuk bayi yang tidak sihat** | **Ya** | **Tidak** | **Tidak tahu** |
| --- | --- | --- | --- | --- |
| 1 | Susu perahan boleh disimpan sehingga 4 jam pada suhu bilik |  |  |  |
| 2 | Susu perahan boleh disimpan sehingga 48 jam di bahagian sejuk biasa bagi peti sejuk dua pintu |  |  |  |
| 3 | Susu perahan boleh disimpan sehingga 2 minggu di bahagian sejuk beku dalam peti sejuk 1 pintu. |  |  |  |
| 4 | Susu perahan boleh disimpan sehingga 3 bulan di bahagian sejuk beku dalam peti sejuk 2 pintu. |  |  |  |

**SEKSYEN C:** **HALANGAN DALAM PEMERAHAN SUSU IBU SEMASA BAYI BERADA DI WAD BAYI (BARRIER ON EXPRESS BREAST MILK WHILE INFANT IN WARD**

| **No Item** | **Halangan dalam pemerahan susu semasa bayi berada di wad bayi** | **Ya** | **Tidak** |
| --- | --- | --- | --- |
| 1 | Saya mempunyai masalah untuk melakukan perahan susu ibu kerana tidak bersedia dengan kelahiran bayi pramatang. |  |  |
| 2 | Perasaan stres dengan kelahiran bayi pramatang mengganggu saya untuk memerah susu. |  |  |
| 3 | Saya berasa tidak selesa semasa mengepam susu. |  |  |
| 4 | Saya mengalami kekurangan privasi semasa memerah susu. |  |  |
| 5 | Saya berasa malu untuk memerah susu menggunakan pam susu |  |  |
| 6 | Saya merasakan perbuatan memerah susu adalah memenatkan. |  |  |
| 7 | Keadaan badan yang keletihan mengganggu saya untuk memerah susu. |  |  |
| 8 | Suami kurang menyokong saya untuk memerah susu |  |  |
| 9 | Keluarga kurang menyokong saya untuk memerah susu |  |  |
| 10 | Saya merasakan kakitangan hospital kurang menyokong saya untuk memerah susu |  |  |
| 11 | Saya tidak mempunyai masa yang cukup untuk memerah susu |  |  |
| 12 | Saya berasa membazir masa untuk memerah susu. |  |  |
| 13 | Memerah susu menyebabkan saya berasa sakit pada payudara |  |  |
| 14 | Pengeluaran susu yang sedikit mengganggu saya untuk memerah susu |  |  |
| 15 | Saya tidak mempunyai pam susu di rumah |  |  |
| 16 | Halangan untuk menghantar susu ke hospital mengganggu saya untuk memerah susu |  |  |

**MAKLUMAT KELAHIRAN DAN BAYI: UNTUK DI ISI OLEH PENYELIDIK**

1. Tarikh bayi di lahirkan:____________________
2. Jumlah minggu semasa bayi dilahirkan (POA):________________minggu
3. Jantina bayi: Lelaki Perempuan
4. Berat lahir bayi: _________________ gram
5. Kaedah bayi dilahirkan:
6. Lahir melalui vagina
7. Lahir secara pembedahan cesarean
8. Lain-lain: Nyatakan___________________

6 . Sebab bayi dilahirkan pramatang. Nyatakan:

1. __________________________________________
2. __________________________________________
3. __________________________________________
4. Masalah kesihatan bayi sekarang. Nyatakan :
5. ___________________________________________
6. ___________________________________________
